# Genetic structure reveals a history of multiple independent origins followed by admixture in the allopolyploid weed *Salsola ryanii*

**DOI:** 10.1111/eva.12399

**Published:** 2016-07-13

**Authors:** Shana R. Welles, Norman C. Ellstrand

**Affiliations:** ^1^ University of CaliforniaRiversideCAUSA

**Keywords:** admixture, allopolyploid, gene flow, hybridization, invasive, *Salsola*

## Abstract

It has recently become clear that many invasive species have evolved *in situ* via hybridization or polyploidy from progenitors which themselves are introduced species. For species formed by hybridization or polyploidy, genetic diversity within the newly formed species is influenced by the number of independent evolutionary origins of the species. For recently formed species, an analysis of genetic structure can provide insight into the number of independent origin events involved in the formation of the species. For a putative invasive allopolyploid species, the number of origins involved in the species formation, the genetic diversity present within these origins, and the level of gene flow between independent origins determines the genetic composition of the neospecies. Here we analyze the genetic structure of the newly formed allopolyploid species, *Salsola ryanii*, a tumbleweed which evolved within the last 20–100 years in California. We utilize the genetic structure analysis to determine that this new species is the result of at least three independent allopolyplodization events followed by gene flow between the descendants of independent origins.

## Introduction

The rate of introduction of non‐native species, including many invasive and weed species, continues to increase annually (Mack et al. 2000) and these introductions have profound impacts on agricultural sustainability and productivity as well as on native biodiversity (Pimentel et al. 2005). For many putative invasive species, postintroduction gene flow plays a major role in the evolution of invasiveness (Blossey and Notzold 1995; Prentis et al. 2008). Gene flow can play a role in the evolution of invasiveness in two distinct ways: (i) new invasive species can form via hybridization between introduced species or an introduced species and a native species (Ellstrand and Schierenbeck 2000) (ii) gene flow (admixture) between populations resulting from independent introduction events from divergent source populations can generate novel combinations of genes and phenotypes which may be important to adaptation during invasion (Keller and Taylor 2010; Verhoeven et al. 2011). There is mixed support for the role of both hybridization (Wolfe et al. 2007; Whitney et al. 2009; Rius and Darling 2014) and admixture in the evolution of invasive species (Whitney et al. 2009); however, for certain newly evolved invasive lineages, it is clear that gene flow between both populations and taxa has played an important role in the evolution of invasiveness (Ellstrand and Schierenbeck 2000; Schierenbeck and Ellstrand 2009; Keller and Taylor 2010).

Of the invasive species that result from hybridization, many have formed via hybridization stabilized by whole genome duplication (allopolyploidy) (Ellstrand and Schierenbeck 2000; Schierenbeck and Ellstrand 2009). For invasive species that evolve via allopolyploidy, the number of independent origin events and whether or not gene flow (admixture) occurs between these origins, shapes the genetic diversity present in the newly formed species and the evolutionary potential of the species (Meimberg et al. 2009).

It was originally believed that allopolyploids consistently result from a single origination event, giving rise to an initially genetically uniform new species (Ownbey and Mccollum 1953; Soltis et al. 1993). Monophylesis appears to have occurred for some allopolyploid lineages (e.g., polyploidy in entire genus of *Gossypium* including the wild polyploids) and in the allopolyploid invasive *Spartina anglica* (Raybould et al. 1991; Rauscher et al. 2004). In contrast, polyphylesis has been demonstrated for many allopolyploid species (e.g., *Tragopogon mirus, Tragopogon miscellus*,* Senecio cambrensis,* and some species in the *Glycine tomentella* complex) (Soltis and Soltis 1991; Ashton and Abbott 1992; Rauscher et al. 2004; Soltis et al. 2004). For newly formed allopolyploid species, one could hypothesize that multiple origins would contribute to the invasiveness of a new allopolyploid, just as multiple introductions can contribute to the evolution of invasiveness in introduced species (Keller and Taylor 2010; Verhoeven et al. 2011). Therefore, determining the number of origins a potentially invasive allopolyploid species is important for assessing its potential invasiveness and for prioritizing control efforts.

### Using population genetics to assess origins and gene flow in an allopolyploid species

In recently formed allopolyploid species, population genetic analyses can be used to estimate the number of origins. Recently formed species have had limited time for the development of population structure beyond the structure generated by the initial origin events. For those taxa, the spatial population genetic structure should thus have a discernable “footprint” of origin events before gene flow and recombination homogenize it beyond recognition. Thus, assessment of number and location of genetic clusters can allow us to estimate the number of species origins and whether dispersal or gene flow between lineages have occurred postpolyploidization in the same way that a population genetic structure analysis can be used to asses number of introduction events (Leak‐Garcia et al. 2013). If a newly formed species is the result of a single origin, we would expect to observe the genetic similarity among all individuals and little genetic structure observed. Any genetic structure that does exist in a single origins species would have evolved following the recent origin and should be significantly less than what is present in the progenitor species, presuming that the progenitor is relatively ancient (≫100 generations) and that the neospecies and the progenitors have similar dispersal abilities. If a newly formed species is the result of multiple independent origins from genetically distinct progenitor populations, the individuals resulting from independent origins will tend to go through a speciation bottleneck and likely be genetically distinct from each other (given typical levels of genetic polymorphism in the ancestral species).

Assuming that independent origin events occur in discrete locations, some spatial genetic structure should be initially present and erode as the different lineages spread, intermix, and potentially admix via propagule, seed, and pollen dispersal. Using a genetic structure model that allows for gene flow can give us insight into (i) potential number of independent origins and (ii) potential early interlineage gene flow. This approach to identifying independent origin events is likely conservative in terms of the number detected because independent origins from parental types with similar genetic compositions are unlikely to be detected. However, this method is extremely unlikely to assign multiple origins when a single origin is the true history.

### Study system

*Salsola ryanii* (2n=54) is a newly formed weedy allohexaploid derivative of *S. tragus* (2n=36) and *S. australis* (2n=16), which formed *in situ* in California in the last 20–100 years (Hrusa and Gaskin 2008). Both of *S. ryanii's* progenitor species are problematic weedy/invasive species; for this reason, studies into the potential invasiveness of the derivative species are crucial. Hrusa and Gaskin (2008) first identified the species in two distinct populations within the Central Valley of California following studies documenting the presence of multiple previously undocumented species or types of *Salsola* in California, USA (Ryan and Ayres 2000; Gaskin et al. 2006). Hrusa and Gaskin (2008) hypothesized that this newly formed species resulted from multiple independent origin events based on the disjunct geographic distribution they observed, but this hypothesis has not been confirmed with genetic data. The range of *S. ryanii* has expanded in recent years and now extends through multiple floristic provinces of California including the Sacramento and San Joaquin Valleys, San Francisco Bay area, Central Coast, Western Transverse Range, and Modoc Plateau (Welles and Ellstrand 2016). The role that new origins have played in the rapid range expansion of *S. ryanii* is unknown.

Neither of *S. ryanii's* progenitors are native to California. *Salsola tragus*' native range extends from North Africa and Western Russia, through Asia into Northeast Siberia and Northeast China*. *The first known introduction of *S. tragus* into North America occurred in South Dakota in the 1870's, likely through contamination of agricultural seed (Young 1988). The number of introductions of *S. tragus* into the US is unknown, but the introduction source was likely Russia (Young 1988). *Salsola tragus* is a highly dispersed species, which spreads seeds by its well‐known “tumbling” strategy, with the whole plant as the diaspore (a.k.a. “chamaechory”). *Salsola australis* is a weed in California and Arizona and is likely native to Australia or South Africa (Borger et al. 2008). *Salsola australis* is morphologically very similar to *S. tragus* and was not recognized as a distinct species until recently (Ryan and Ayres 2000). Given that *S. australis* was an unrecognized cryptic species assigned to *S. tragus* until recently, little is known of the introduction of *S. australis* into North America.

This is a preliminary study of the origins of *S. ryanii*. The range of this species has increased dramatically following its formation; however, it has a smaller range than its progenitors at the time of this study (Welles and Ellstrand 2016), which limited our ability to sample *S. ryanii* broadly and evenly. In this preliminary study, we (i) determine whether *S. ryanii* is the result of single or multiple origin events utilizing an analysis of its genetic structure and (ii) investigate patterns of dispersal and admixture that have occurred following its origin(s).

## Materials and methods

### Sampling

Plant samples were collected throughout the range of *S. ryanii* in California as described in (Welles and Ellstrand 2016). A total of 135 *Salsola ryanii* individuals were collected from 25 locations, 165 individuals of *S. tragus* were sampled from 20 locations, and 226 individuals of *S. australis* were sampled from 25 locations. All collection locations were at least 16 km apart from each other; multiple species were often sampled from the same location. The location of each collection was noted using a Garmin GPS map 62s unit.

### DNA extraction and microsatellite analysis

DNA was extracted from each sample using a modified CTAB extraction method, with the incubation step following grinding with liquid nitrogen omitted (Doyle 1991). The species of each sample was identified using species‐specific intersimple sequence repeat (ISSR) markers for each of the progenitor species; collections were identified as the allopolyploid species *S. ryanii* if they contained all species‐specific bands, as described in (Welles and Ellstrand 2016). DNA was amplified using five microsatellite primers (CT4, CT6, SB15, SB09, BMB3) developed for *Salsola* species by (McGray et al. 2008). Polymerase chain reaction (PCR) was carried out according to protocols established by McGray et al. (2008). Following amplification samples were analyzed using the ABI 3130XL Genetic Analyzer. Alleles were binned using GeneScan (ABI). For the two polyploid species, when fewer than the number, potential number of unique alleles were detected (6 for *S. ryanii* and 4 for *S. tragus*) and all the remaining alleles were coded as missing data because in polyploid species it is not possible to know which allele is present in multiple copies or a single copy. For the diploid species (*S. australis*), when only one allele was detected it was coded as homozygous locus. This does potentially code null alleles as a homozygous loci.

### Genetic structure analysis

Population structure of all three species (*S. ryanii, S. tragus* and *S. australis*) was estimated using STRUCTURE (Pritchard et al. 2000). Although five loci is a relatively small number of loci for this type of analysis, multiple other studies have done the same type of analyses with a similar number of loci (Culley and Wolfe 2001; O'Leary et al. 2007). The population structure of each species was analyzed independently using a 10 000 burn‐in period, 50 000 MCMC replicates following the burn‐in period and a model including admixture. The appropriate number of genetic clusters was determined using a nonparametric Wilcoxon test (Rosenberg et al. 2001) and confirmed using second‐order rate of change of likelihood for lineages where greater than one genetic cluster is determined as the optimum number of clusters by the Wilcoxon test with 10 repetitions for each species (Evanno et al. 2005).

## Results

**Figure 1:**
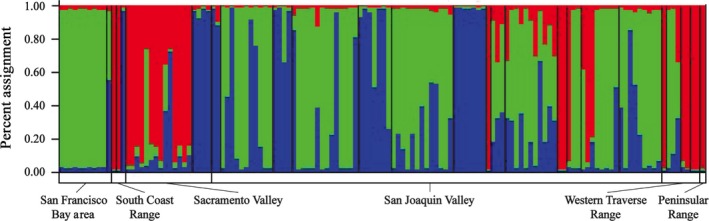
STRUCTURE plot for *S. ryanii*. Black lines within the plot separate collections, each made a minimum of 16 km apart. The bars on the bottom represent different floristic regions. Within floristic provinces collections are organized north to south.

The optimal number of genetic clusters (K) was 3 for *S. ryanii*, 2 for *S. australis* and 1 for *S. tragus*. In this case, the allopolyploid species, *S. ryanii,* has the largest number of genetic clusters of the three species. Figure 1 and Table 1 show three genetic clusters of *S. ryanii* that are geographically dispersed throughout its range, with each of the genetic clusters occurring in multiple floristic provinces.

**Table 1:** Summary of STRUCTURE analysis (Fig. 1) for *S. ryanii*. Cluster A is the green cluster, B is the blue cluster, and C is the red cluster in Fig. 1. Collections presented in the same order as in Fig 1, divided by floristic province and presented North to South within each province (Baldwin and Goldman, 2012)

Collection	# of individuals	% Cluster A (#)	% Cluster B (#)	% Cluster C (#)	% admixed (#)	GPS Coordinates
SFBA 1	10	100% (10)	0	0	0	37°21.829′N 121°74.175′W
SFBA 2	1	0	0	0	100% (1)	36°99.548′N 121°56.318′W
SFBA Total	11	91% (10)	0	0	9% (1)	
SCR 1	1	0	0	100% (1)	0	34°34.464′N 119°07.656′W
SCR 2	2	0	0	100% (1)	0	34°75.321′N 119°40.256′W
SCR Total	3	0	0	100% (2)	0	
SCV 1	1	0	100% (1)	0	0	39°35.724′N 121°4.031¢W
SCV 2	14	0	0	43% (6)	57% (8)	39°00.368′N 121°4.022′W
SCV 3	4	0	100% (4)	0	0	39°40.243′N 121°4.125′W
SCV Total	19	0	26% (5)	32% (6)	42% (8)	
SJV 1	2	0	100% (2)	0	0	38°45.4332′N 121°40.265′W
SJV 2	11	55% (6)	9% (1)	0	36% (4)	38°11.531′N 121°39.420′W
SJV 3	4	75% (3)	0	0	21% (1)	37°30.400′N 120°50.417′W
SJV 4	14	64% (9)	7% (1)	0	29% (4)	36°99.515′N 120°10.641′W
SJV 5	7	0	71% (5)	0	29% (2)	36°73.878′N 119°89.739′W
SJV 6	13	46% (6)	0	0	54% (7)	35°60.028′N 119°89.739′W
SJV 7	7	0	100% (7)	0	0	36°40.243′N 119°90.016′W
SJV 8	1	0	0	100% (1)	0	36°32.743′N 119°91.154′W
SJV 9	3	0	0	0	100% (3)	36°17.156′N 119°92.546′W
SJV 10	11	18% (2)	0	0	82% (9)	36°05.341′N 120°06.976′W
SJV 11	2	0	0	100% (2)	0	35°82.241′N 119°90.9241′W
SJV 12	3	66% (2)	0	33% (1)	0	35°05.341′N 120°06.976′W
SJV 13	8	50% (4)	0	0	50% (4)	35°62.176′N 119°44.583′W
SJV 14	9	56% (5)	0	0	46% (4)	35°36.466′N 119°34.817′W
SJV Total	95	37% (35)	17% (16)	3% (3)	40% (38)	
WTR 1	1	0	0	100% (1)	0	35°07.582′N 118°24.865′W
WTR 2	3	33% (1)	0	0	66% (2)	34°30.733′N 118°32.070′W
WTR 3	2	0	0	100% (2)	0	34°10.541′N 118°320.172′W
WTR 4	2	0	0	100% (2)	0	33°81.260′N 118°45.149′W
WTR Total	8	13% (1)	0	63% (5)	25% (2)	
PR 1	2	0	0	100% (2)	0	33°42.463′N 116°14.949′W
*S. ryanii* Total	135	34% (46)	16% (21)	13% (18)	36% (49)	

SFBA, San Francisco Bay Area; SCR, South Coast Range; SCV, Sacramento Valley; SJV, San Joaquin Valley; WTR, Western Traverse Range; PR, Peninsular Range.

We considered an individual admixed if its assignment to any single cluster did not exceed 90% (Table 1), we used 90% as opposed to the 95% that has been suggested in other studies (Blair and Hufbauer 2010) because making a conservative estimate of admixture ensures that admixture is not overestimated. In this study, 36% of the 135 total *S. ryanii* individuals appear to be admixed; of the nonadmixed individuals (64%) are assigned to one of the three genetic clusters. The green cluster (A) of Fig. 1 is represented by the most individuals (34%), the other two clusters are roughly equal to each other in frequency, 16% and 13%, respectively (Table 1). The Sacramento and San Joaquin Valleys have the largest percentage of admixed individuals; however, all of the floristic provinces with the exception of the South Coast Range and the Peninsular Range have some admixed individuals (Table 1).

**Figure 2:**
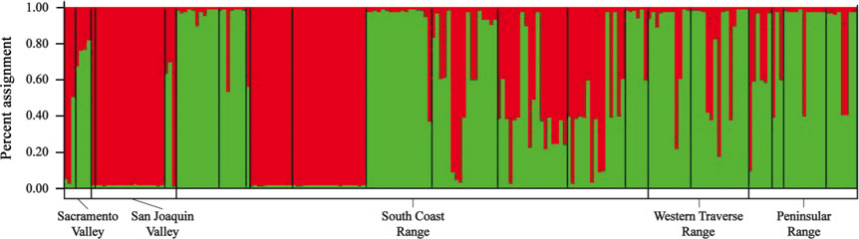
STRUCTURE plot for *S. australis*. Black lines within the plot separate collections each made a minimum of 16 km apart. The bars on the bottom represent different floristic regions. Within floristic province collections are organized north to south.

We did not detect any population structure for the tetraploid parent *S. tragus*. We do not present a STRUCTURE plot for *S. tragus* because a STRUCTURE plot with *K*=1 is uninformative. For *S. australis,* the optimal number of genetic clusters was 2, with both clusters occurring in each of the floristic provinces (Fig. 2). In the Sacramento Valley, San Joaquin Valley, and South Coast Range (Central California Provinces) there is limited admixture, with most individuals being assigned to a single genetic cluster (Fig. 2). The San Joaquin Valley was the area with the largest number of individuals and it had the largest proportion of variable loci, along with the Sacramento Valley, and the largest number of private alleles (Table 2). The South Coast, Peninsular Range and Western Transverse Range (Southern California Provinces) largely contain admixed individuals (Fig. 2).

**Table 2:** Summary statistics for *S. ryanii* populations

Region	# of individuals	Proportion of variable loci	# of Private Alleles
SFBA	11	0.2	0
SCR	3	0.6	4
SCV	19	1	6
SJV	95	1	19
WTR	8	0.8	2
PR	2	0.4	0

SFBA, San Francisco Bay Area; SCR, South Coast Range; SCV, Sacramento Valley; SJV, San Joaquin Valley; WTR, Western Traverse Range; PR, Peninsular Range (Baldwin and Goldman, 2012).

## Discussion

The genetic structure patterns that we observe for the newly formed allopolyploid species *S. ryanii* are consistent with the expectations of a species formed through multiple independent origins. Based on our data, it appears that there are at least three origins of *S. ryanii* that have subsequently dispersed and recombined to generate the observed genetic structure. The discovery of multiple origins of *S. ryanii* is consistent with prior the predictions of Hrusa and Gaskin (2008) and with the polyphyletic origins of multiple other invasive allopolyploid species (Soltis and Soltis 1991; Ashton and Abbott 1992; Soltis et al. 2004). Given that only five microsatellite markers were used in this analysis the results must be interpreted accordingly. It is likely that there is additional genetic structure present within some or all of the species that is cryptic given this marker density. Future work should confirm these results with a more in‐depth analysis and an increased marker density.

In addition to the assignment of multiple genetic clusters, our results contain other information that reinforces the hypothesis of multiple origins. First, we detected more clusters in *S. ryanii* than for either progenitor. A single origin would likely lead to a single cluster and, even with complex backcrossing, no more than what is observed in the most structured progenitor species, whose cluster assignment is only affected by mutation and gene flow. Second, in *S. ryanii* we observe the three genetic clusters more‐or‐less scattered throughout its current range. The geographic intermixing of genetic clusters of *S. ryanii* is what would be expected in the event of multiple independent origin events followed by dispersal. We would not expect such a pattern to occur in such a short time after a single origin event followed by dispersal and subsequent evolution of population structure. In the latter case, we would expect that the genetic signature of a single of origin should still be apparent. Hybridization and genome duplication could spur rapid evolution following polyploidization; however, if this rapid evolution were to generate population structure following a single origin, we would expect genetically similar populations to be spatially segregated.

The high‐level of dispersal in *S. ryanii* that is apparent from the geographic distribution of the genetic structure analysis is consistent with the dispersal abilities of the progenitor species and with previous work demonstrating that the range of *S. ryanii* has expanded rapidly and dramatically as its geneses (Welles and Ellstrand 2016). The observed lack of geographic differentiation of *S. tragus* found in this study is also consistent with expectations for a species with very high dispersal ability (Bohonak 1999). *Salsola australis* also has appears to have some level of wind dispersal but lacks both the round plant shape and the formation of an abscission layer at the base of the plant following fruit maturity that make *S. tragus* a highly dispersed species (S. Welles, Personal observation). The morphological differences are consistent with the differences in population structure observed; we found that *S. australis* has some limited population structure but more structure than is observed in the closely related highly dispersing species *S. tragus*.

Given the substantial fraction of admixed *S. ryanii* individuals detected, it appears that gene flow has occurred between descendants of the multiple independent origins. We also observe that multiple *S. ryanii* lineages are currently located within the same or nearby collection sites, creating the potential for additional gene flow in the future. The genetic structure of *S. ryanii* contrasts with what has been found for the multiple allopolyploid lineages of *Tragopon* (*T. mirus* and *T. miscellus*) which are the result of multiple origins but do not admix with each other (Soltis et al. 2004). Given the patterns we observed and the morphological similarity between *S. ryanii* and *S. tragus* (Hrusa and Gaskin 2008), we predict that, in the absence of any future novel lineages of *S. ryanii*, the population genetic structure of *S. ryanii* is likely to homogenize and eventually converge similar to *S. tragus*.

### Application and future directions

Our finding of multiple origins followed by dispersal and gene flow together, with previous studies demonstrating increased fitness and a rapid range expansion (Welles and Ellstrand 2016), suggests a strong potential for *S. ryanii* to become a problematic weed species. Future work should address the biology of this newly formed species to determine how problematic a weed this species will be as its range continues to expand and what the best management practices are for this species. The finding of multiple origins followed by admixture suggests that this species is potentially better able to adapt in response to control methods compared with a species descended from a single origin or independent origins that are not able to cross with one another.

Future work should compare the fitness of *S. ryanii* to its progenitors as well. Fitness experiments should also address as whether fitness differences exist between populations that descend from a single origin versus populations that descend from multiple origins. Additionally, selection experiments should be conducted to confirm that populations formed via admixture between independent origin events have an enhanced ability to respond to selection. These experiments in addition to previous studies showing rapid range expansion in *S. ryanii* will allow for prioritization of *Salsola* species and populations for control methods and will also allow for prioritization of populations that require careful integrated management techniques to prevent adaptation to control methods. *Salsola ryanii* and its progenitors are an ideal system to understand the importance of gene flow to the evolution of invasive species. Fitness experiments between *S. ryanii* and its progenitors can test the role of hybridization (gene flow between species), while fitness experiments between individuals derived from single versus multiple origins allows for a test of the role of postpolyploidization gene flow to the evolution of invasiveness.

## Data archiving statement

Data available from the Dryad Digital Repository: http://dx.doi.org/10.5061/dryad.k28r8.
